# Influence of the Anesthetic Technique on Circulating Extracellular Vesicles in Bladder Cancer Patients Undergoing Radical Cystectomy: A Prospective, Randomized Trial

**DOI:** 10.3390/cells12202503

**Published:** 2023-10-23

**Authors:** Luisa Gluth, Crista Ochsenfarth, Phuong Nam Viet Pham, Jan M. Wischermann, Thomas Komanek, Florian Roghmann, Ulrich H. Frey

**Affiliations:** 1 Department of Anesthesiology, Intensive Care, Pain and Palliative Care, Marien Hospital Herne, Ruhr-University Bochum, 44801 Bochum, Germany; luisa.gluth@elisabethgruppe.de (L.G.);; 2 Department of Urology, Marien Hospital Herne, Ruhr-University Bochum, 44801 Bochum, Germany

**Keywords:** extracellular vesicles, miRNAs, bladder cancer, anesthetics

## Abstract

Anesthetics have been shown to alter tumor progression and seem to influence surgical cancer outcome. Circulating extracellular vesicles as mediators of intercellular communication are involved in cancer progression and may be influenced by anesthetics. In this prospective, randomized study, effects of anesthetics on extracellular vesicles and associated micro-RNAs in bladder cancer patients undergoing radical cystectomy were tested. Extracellular vesicles from 51 patients at four perioperative time points receiving Propofol or Sevoflurane were extracted with polymer-based methods and quantified with a nanoparticle-tracking analysis. Vesicle-associated micro-RNAs were analyzed with a real-time polymerase chain reaction using array cards and single assays for tumor-associated miR-21-5p, miR-15a-5p, miR-17-5p and miR-451a. Plasma extracellular vesicle concentration (suture: fold change (fc) in Propofol at 4.1 ± 3.9 vs. Sevoflurane at 0.8 ± 0.5; *p* = 0.003) and associated miRNAs increased significantly (+30% post induction, +9% 30 Min surgery) in the Propofol group. Tumor-associated miRNAs increased during surgery in both groups (fc in miR-21-5p: 24.3 ± 10.2, *p* = 0.029; fc in miR-15a-5p: 9.7 ± 3.8, *p* = 0.027; fc in miR-17-5p: 5.4 ± 1.7, *p* = 0.014), whereas antitumor miR-451a increased in the Propofol group only (fc: 2.5 ± 0.6 vs. 1.0 ± 0.2; *p* = 0.022). Anesthetics influence extracellular vesicles and associated micro-RNAs of bladder cancer patients during surgery. Increased expression of antitumor micro-RNA may be an explanatory approach for decreased tumor cell viability after Propofol.

## 1. Introduction

Bladder cancer takes 10th place among cancer cases worldwide and the average 5-year survival rate in Europe is 70% [1]. Radical cystectomy (RC) is regarded as the therapeutic standard for muscle-invasive carcinomas and can be performed as open or laparoscopically robot-assisted, whereas both methods are comparable in terms of progression-free survival [2]. Nevertheless, every fourth bladder cancer patient suffers from distant metastases within 2 years after RC [3]. Surgery itself is considered a risk for intraoperative tumor cell dissemination [4] and can lead to reactive inflammation and altered immune response, thereby potentially enabling growth and adhesion of the tumor cells in the patient’s blood circulation [5]. Anesthetics are considered to have an influential role in promoting tumor progression [6,7]; moreover, numerous studies indicate differences in outcome after cancer surgery [8]. Propofol seems to be beneficial in terms of recurrence-free survival, especially for bladder cancer [6,9,10], which underscores the importance of this topic for further research at molecular levels [11]. Micro-RNAs (miRNAs) from extracellular vesicles (EVs) seem to play a major role as targets of anesthetics [12,13]. EVs are particles surrounded by a lipid bilayer and represent an important intercellular signal transmitter [14]. They are present in all human body fluids and can be classified into three subtypes [15]: apoptotic bodies (1–5 µm), which emerge during controlled cell death; microvesicles (100–1000 nm), which form by budding from the plasma membrane; and exosomes (50–100 nm), which grow intracellularly from multivesicular bodies and are released with exocytosis [16]. EVs represent biologically active molecules that reflect the degree of differentiation of their cell of origin and are dysregulated in various cancers [17,18]. EV-associated miRNAs can be modulated by various stimuli, among others, and also by anesthetics. While in cancer surgery outcome differences have been described depending on the anesthetic method, the influence of the anesthetics on the EV-associated miRNAs may possibly play an important role here. However, studies on EV-mediated effects of anesthetics are sparse with little research on volatile anesthetics and the effect of these anesthetics on miRNAs in urinary bladder carcinoma is poorly understood. The primary aim of this study was therefore to investigate the influence of Sevoflurane and Propofol on size and concentration of circulating EVs in bladder carcinoma patients during RC. The second aim was to determine whether there are changes in miRNA signatures of EVs depending on the anesthetic procedure during RC. For this purpose, EV characteristics and miRNA signatures from EVs were analyzed in relation to the anesthetic procedure during surgery in a prospective randomized trial.

## 2. Materials and Methods

### 2.1. Study Design

Patients with a malignant neoplasm of the urinary bladder, who underwent radical cystectomy at Marien Hospital Herne, University Hospital of the Ruhr-University Bochum, Germany, were included in this prospective, controlled, randomized, partially blinded study after detailed information and written informed consent. The study was approved by the Ethics Committee of the Ruhr-University Bochum (Registration No.: 20-7027-NIS) on 11 September 2020, and the study was registered in the German Registry for Clinical Studies (DRKS-ID: DRKS00023682) on 25 November 2020. After checking for eligibility block-wise (10 patients per block, 6:4 randomization), randomization was performed in a standardized manner using consecutively numbered envelopes during the anesthesiological informed consent interview by physicians and data were collected and stored using REDCap (Research Electronic Data Capture), which is a secure, web-based software platform designed to support data capture for research studies [19]. While this was a pilot study, no case number calculation was performed. For more valid results, we planned to include a total of 100 patients in this study. Over a period from December 2020 to September 2021, 87 patients were screened for eligibility. Patients were assigned parallel to two intervention groups, anesthesia with Sevoflurane (Sevo) as balanced anesthesia or Propofol (Prop) as total intravenous anesthesia, in an allocation ratio of 1:1. Both therapies represent established treatment options, so that no disadvantages for the patients were to be expected with either of the two anesthetic procedures. Patients, informing physicians, as well as anesthesiologists present during the operation were informed about the procedure of anesthesia, whereas the laboratory physicians were blinded until all experiments were analyzed.

**Figure 1:**
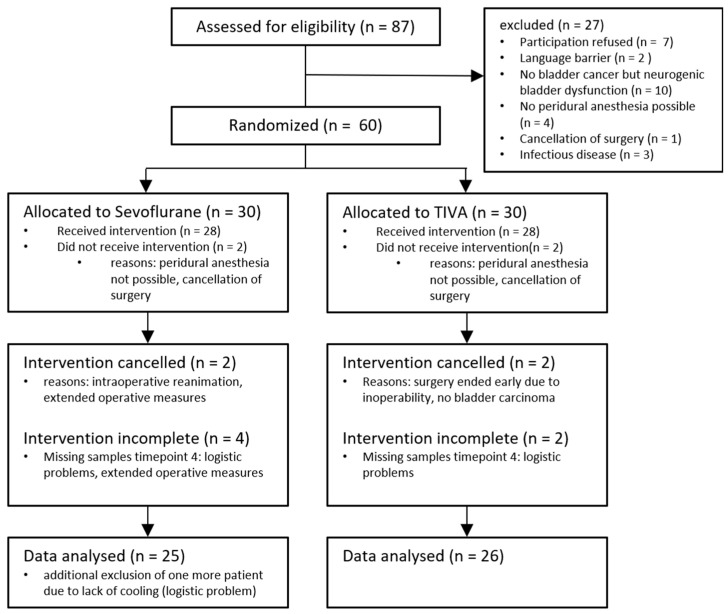
CONSORT flowchart for the EXoRC trial. A total of 87 patients were screened, of which 60 patients were randomized. Ultimately, 25 patients of the Sevoflurane group and 26 patients of the Propofol group were included in the analysis.

Recruitment was based on defined inclusion and exclusion criteria. All patients had a pathologically histologically confirmed bladder carcinoma and were about to undergo radical cystectomy with urinary diversion. A minimum age of 18 years, adherence to the Enhanced Recovery After Surgery (ERAS) protocol and assignment to the American Society of Anesthesiologists (ASA) anesthesiologic risk score of classes II/III and epidural anesthesia in combination with standard anesthesia were considered as inclusion criteria. Exclusion criteria were being under the minimum age of 18 years, a language barrier and clinically relevant aortic stenosis. A total of 60 patients were randomized and a total of 51 patients were included in the final analysis, whereas all patients are listed according to the consolidated standards of reporting trials (CONSORT) requirements [20] (see Figure 1, showing the CONSORT flowchart).

### 2.2. Study Protocol

Study participants were recruited according to the inclusion and exclusion criteria. All anesthetic-assisted procedures were performed in a standardized manner. Awake patients received an arterial catheter under local anesthesia for invasive blood pressure measurement and blood collection. After sterile placement of a peridural catheter (Th 9–12), followed by a test dose of 3 mL of 0.75% ropivacaine, the electroencephalogram (EEG; Narcotrend, Hannover, Germany) and an advanced blood pressure monitoring system (Acumen Hypotension Prediction Index, Edwards Lifesciences Corp., CA, USA) were applied. Anesthesia was intravenously induced using fentanyl (2–4 µg/kg), Propofol (2–4 mg/kg) and muscle relaxation using rocuronium (0.6–0.9 mg/kg) or succinylcholine (1 mg/kg). According to the ERAS protocol, single intravenous administration of 2 g of ceftriaxone and 500 mg of metronidazole was given approximately 30 min preoperatively. In the anesthetized patient, a central venous catheter (CVC) was established. In the TIVA group, a Propofol perfusor was connected after intravenous induction of anesthesia. Propofol run rates were adjusted individually, depending on the EEG, with an anesthetic depth index target of 45. In the Sevoflurane (Sevo) group, Sevo was adjusted to reach a minimum alveolar concentration (MAC) of 0.8 to 1.2 and an anesthetic depth index target value of 45. To check whether other drugs may influence the expression of extracellular vesicles, statistical analyses were performed determining whether one group was exposed to significantly increased doses of medication. Next to intraoperative characteristics, patient data such as age, ASA status, neoadjuvant treatment of bladder cancer and others were considered.

Four arterial blood samples of 7.5 mL of EDTA plasma blood each (Sarstedt AG & Co. KG, Nümbrecht, Germany) were obtained during routine perioperative blood sampling. After placement of the arterial catheter for invasive blood pressure measurement, the first sample was taken via the radial artery while the patient was awake (baseline). Additional arterial blood samples were collected after CVC placement in anesthesia (anesthesia), 30 min after incision (30 min surgery) and after surgical suture (surgical suture). Samples were immediately stored at 4 °C for a maximum of 2 hours and then centrifuged at 4 °C and 2000 G for 15 min. At each time point, EDTA plasma samples were aliquoted into six LoBind tubes (Eppendorf AG, Hamburg, Germany), each containing 500 µL, labeled and snap-frozen in liquid nitrogen. Samples were stored at −80 °C until further processing.

### 2.3. Extraction of Extracellular Vesicles

EVs were precipitated and extracted using a polymer-based procedure [21] using 500 µL aliquots of EDTA plasma samples and the ExoQuick-TC^TM^ Exosome Precipitation Solution (System Biosciences, Palo Alto, USA) as recommended by the manufacturer. After resuspension of the pellet, remaining polymers were removed by filtering samples using Cytiva PD SpinTrapTM G-25 (Merck KgaA, Darmstadt, Germany) (see procedural instructions, supplemental digital content S1, extraction of extracellular vesicles, for details of the experiment protocol).

### 2.4. Western Blot Analysis

First, quantitative protein determination was performed using the Pierce^TM^ BCA Protein Assay Kit (Thermo Fisher Scientific, Rockford, USA). In total, 50 µL of EVs was mixed one-to-one with an RIPA-PI buffer followed by vortexing and centrifugation. The supernatant was transferred to a DNA LoBind tube. Efficacy of EV extraction was checked using examination of putative EV-free EDTA plasma supernatants. Next, 4–20% polyacrylamide gels (Mini-PROTEANR TGX^TM^ Precast Gels, Bio-Rad Laboratories GmbH, Munich, Germany) were prepared and 15 µg of protein of the lysate samples was added with human leukemia cell line HL60 (whole cell lysate, ab7914, abcam, Berlin, Germany), and human epithelial cells of a cervical carcinoma, HeLa, as positive controls. Electrophoretic transfer from the gel onto a nitrocellulose membrane was performed using a transfer cell (Trans-BlotR SD Semi-Dry Transfer Cell, Bio-Rad Laboratories, Hercules, CA, USA). The following primary antibodies were used: ALIX (1A12) Mouse monoclonal IgG1 (sc-53540); CD63 mouse monoclonal IgG2a (MX-49.1295.5); Flotillin-1 mouse monoclonal IgG (C-2) (sc-74566; all Santa Cruz Biotechnology, Inc., Heidelberg, Germany). The chemiluminescent reaction was detected in the Molecular Imager R ChemiDocTM XRS+ Imaging System (Bio-Rad Laboratories GmbH, Munich, Germany).

### 2.5. Nanoparticle Tracking Analysis

The Particle Tracking Analyzer NTA ZETAVIEW^®^ (Particle Metrix GmbH, Meerbusch, Germany) was used for the analysis of EVs. A Nanoparticle Tracking Analysis (NTA) is based on a video analysis of the Brownian molecular motion of different particles. Particles are irradiated with a laser and their scattered light is detected using an ultra-microscope so that particle size and concentration can be determined [21]. The samples were diluted until a particle count in the optimal measurement range between 50 and 200 particles per position was reached within the measurement cell. A measurement procedure included five cycles with eleven measurement positions. Only samples for which at least eight positions were recommended for use were included in the evaluation. The “region of interest” (ROI) was set to 50–150 nm [22] according to the expected size of exosomes.

### 2.6. miRNA Extraction from Extracellular Vesicles

The mirVANA^TM^ miRNA Isolation Kit (Invitrogen, Thermo Fisher Scientific, Waltham, MA, USA) was used to isolate miRNAs from EVs. Diluted RNAse A (Thermo Fisher Scientific, Waltham, MA, USA) was added to the EVs to degrade RNAs outside of EVs, thus preventing possible contamination with non-EV-associated miRNAs. cel-miR-39-3p (Invitrogen, Thermo Fisher Scientific, Waltham, MA, USA) was added as a reference to calibrate the samples (spike-in). The RNA samples were stored at −80 °C until further use.

### 2.7. Gene Expression Analyses

For a quantitative real-time polymerase chain reaction (PCR), miRNAs were transcribed into cDNAs using the TaqMan™ Advanced miRNA cDNA Synthesis Kit (Applied Biosystems, Waltham, MA, USA) using a poly-(A) tail and universal random primers. To provide detection and quantification of miRNAs for follow-up experiments, miRNA arrays were performed. For this purpose, the TaqMan^®^ Array Human miRNA A Card v2.0 (ThermoFisher Scientific, Waltham, MA, USA) was used. Using one array card, 377 miRNA targets and 4 controls can be detected simultaneously. Samples of miRAmp reaction of cDNA transcription from RNAs were thawed, vortexed and briefly centrifuged followed by adding cDNA samples diluted at 1:5 with a 0.1X TE buffer. The assays were performed as recommended by the manufacturer and analyzed using Expression Suite Software (Version 1.0.4, Thermo Fisher Scientific, Waltham, MA, USA). Single quantitative real-time polymerase chain reactions were performed using a QuantStudio^TM^ 7 Flex Real-Time PCR System (Applied Biosystems, Waltham, MA, USA) using the following assays: Hsa-miR-15a-5p (477858), Hsa-miR-17-5p (478447), Hsa-miR-21-5p (44975), Hsa-miR-451a (478107), Cel-miR-39-3p (478293; all from Thermo Fisher Scientific Inc., Waltham, MA, USA).

### 2.8. Statistics

Metric data were tested for normal distribution using Kolmogorov–Smirnov tests and reported as the mean ± standard deviation in case of normal distribution; otherwise, the median and interquartile range (IQR) were reported. In the case of normal distribution, groups were tested using a two-tailed independent paired or unpaired t-test; in the case of non-normally distributed variables, a Mann–Whitney U test was used. Categorical variables were expressed as the number and percent and analyzed using the chi-square test. For numbers of cases less than five per group, a Fisher’s test was used. Demographic and intraoperative data were extracted via the reporting system of Philips IntelliSpace and Critical Care and Anesthesia (ICCA, Philips Medical Systems, Andover, MA, USA). For each patient, a full dataset was generated via the SQL-database in the environment of Microsoft SQL Server Management Studio (version 18.7.1, Washington, DC, USA). qPCR assays were evaluated using the delta-CT method [23] and relative miRNA expression was calculated using the spike-in cel-miR-39-3p as a control. The significance level was set at α = 0.05. All statistical analyses were carried out with SPSS software (Version 26, IBM, Armonk, NY, USA) or Graph-Pad Prism 9 (GraphPad Software, San Diego, CA, USA).

## 3. Results

The anesthetic regimen seems to influence cancer surgical outcome. EVs as important mediators of intercellular communication may play a role in tumor progress as possible targets of anesthetics that have been used during surgery. It has been shown that Propofol inhibits cancer proliferation in bladder cancer cells and other cancer entities [24,25]. On the other hand, inhalational anesthetics, such as Sevoflurane, are associated with increased cancer progression and enhanced development of metastasis in bladder cancer cells [26]. Additionally, outcome studies investigated an enhanced disease-free survival of bladder cancer patients after combined anesthesia with Propofol compared to patients who received Sevoflurane and opioids [10]. Retrospective analyses of patients with different cancer types receiving inhalational anesthesia compared with intravenous anesthesia with Propofol during surgical cancer resection showed a beneficial overall survival for patients who received Propofol during surgery [8]. As other studies show no difference between inhalational and intravenous anesthesia [27], the effect of anesthetics on a biomolecular base on bladder cancer patients is not yet properly elucidated. To enhance knowledge on the influence of anesthetics on EVs, we conducted a randomized, prospective analysis of bladder cancer patients receiving either Propofol or Sevoflurane during radical cystectomy and present our results in the following.

### 3.1. Demographics

Fifty-one patients completed the protocol and were included in the final analysis. Considering demographic data, there were significantly more women in the Sevoflurane group than in the Propofol group (Sevo: 32%, Prop: 8%, *p* = 0.038; Table 1). Intraoperatively, a significantly increased use of the vasopressor norepinephrine (*p* = 0.03) was observed in the Sevoflurane group. Regarding pre-existing conditions and other patient characteristics, as well as all other intra- and postoperative characteristics, the groups were shown to not be significantly different and thus comparable (Table 1).

### 3.2. Validation of the EV Extraction Method

**Figure 2:**
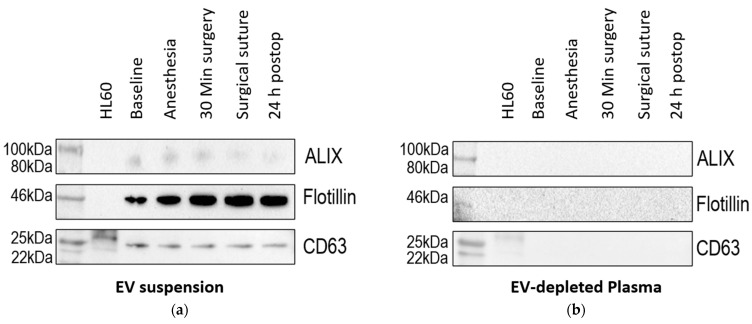
Western blot: Testing for EV-characteristic proteins ALIX, flotillin and CD63. Extracted EVs show positive bands for EV-characteristic proteins (**a**). The EV-free plasma shows no bands (**b**).

To validate the extraction method, Western blots were performed to detect EV-typical proteins (Figure 2). With electrophoretic separation of the proteins of the putative EV samples, the presence of the EV-typical proteins CD63, ALIX and flotillin could be detected (Figure 2a). The absence of those typical markers in the EV-free plasma demonstrated the efficacy of the extraction method (Figure 2b).

Currently, there are no specific singular EV markers for EV characterization but the International Society of EVs recommends the use of cell-membrane-dependent and transmembrane proteins like CD63, CD81 or CD82 or cytosolic proteins like ALIX and flotillin, which are connected to the endosomal sorting complex required for transport [15]. As the presence or absence of EV markers is dependent on the cell of origin [28], it has to be remarked that EV markers vary between EV subpopulations and that EV analyses always contain different subtypes of EVs [29].

### 3.3. EV Size and Concentration

**Figure 3:**
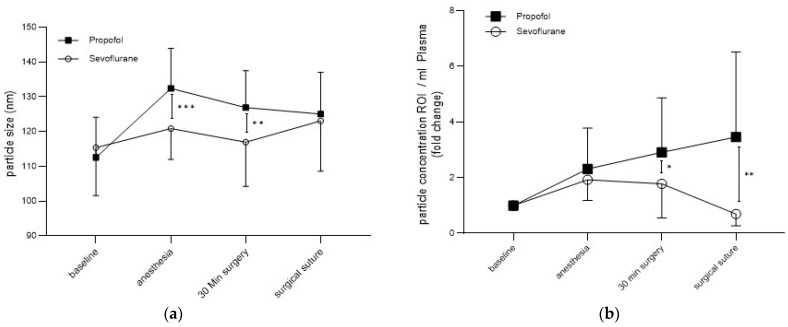
Mean measured particle size and concentration. Basal, after induction of anesthesia, 30 min surgery time and at suture. Mean ± SD. Size (**a**), concentration (**b**). * *p* < 0.05, ** *p* < 0.01, *** *p* < 0.001.

To test the EVs for size and concentration, a Nanoparticle Tracking Analysis (NTA) was performed. From the NTA measurements for the entire patient population, the average basal particle size was 113 ± 12.7 nm. Comparison of randomization groups revealed that the mean particle size initially increased in both groups after induction of anesthesia (Sevo: 120 ± 8.87 nm, Prop: 132 ± 11.5 nm) and decreased slightly after the 30 min surgery time (Sevo: 116 ± 12.7, Prop: 126 ± 10.6 nm), with a statistically significant difference between Sevo and Prop observed for both time points (anesthesia: *p* = 0.0002; 30 min surgery time: *p* = 0.005; Figure 3a). At the end of surgery, particle sizes were similar again (Sevo: 123 ± 14.4; Prop: 125 ± 12; *p* = 0.66).

In addition to size, anesthesia-related changes of particle concentration were of particular interest to uncover potential effects of anesthetics on EVs. For this purpose, particle concentrations were measured in the size range of 50 to 150 nm (ROI), corresponding to the expected exosomal size, to approximately differentiate EV subtypes. In the Sevoflurane group, there was initially a significant increase in particle concentration after induction of anesthesia and 30 min after incision (fc in anesthesia: 1.9 ± 0.7, *p* <0.0001; fc in 30 min surgery: 1.8 ± 1.2; *p* = 0.005), with a significant decrease in particle concentration at suture compared to basal values (fc in suture: 0.7 ± 0.4, *p* = 0.016). In the Propofol group, however, there was a constant increase in particle concentration during surgery, which was significant at all time points (fc in anesthesia: 2.3 ± 1.5, *p* = 0.0001; fc in 30 min surgery = 2.9 ± 2.0, *p* < 0.0001; fc in suture = 3.6 ± 3.1, *p* = 0.002). Comparison of both groups at different time points revealed significantly different particle concentrations between the Sevoflurane and the Propofol group 30 min after incision (*p* = 0.02) and towards suture (*p* = 0.001; Figure 3b). In conclusion, depending on the anesthetic procedure, significantly different particle sizes and concentrations occur during surgery.

### 3.4. Detection of miRNA Expression from EVs with Array Analyses

To verify whether the concentration changes were associated with altered EV content, miRNA expressions of the EVs were examined. For this purpose, the EV array analysis was performed for the first 10 consecutive patients (5 Sevo vs. 5 Prop), in which samples were analyzed for 384 known miRNAs. For comparability, the demographic data of the 10 patients were evaluated, and the two groups were found to be comparable in terms of patient characteristics, bladder cancer classification, previous diseases and intraoperative and postoperative data (see table, supplemental digital content S2, showing the demographic data of the patients for array analyses).

**Figure 4:**
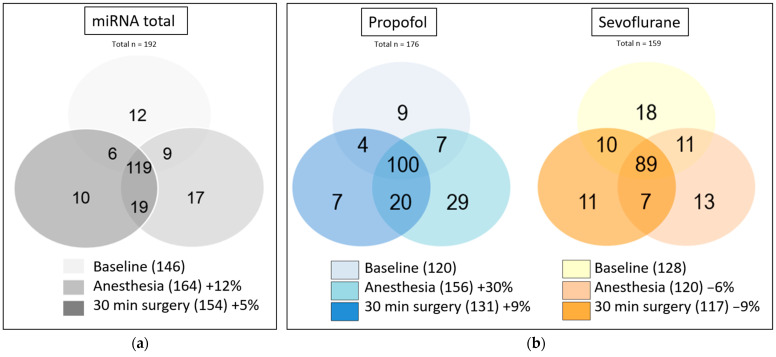
miRNA expression patterns of array studies in Venn diagrams. MiRNA expression of the 10 patients, regardless of the anesthetic procedure. Among others, four tumor-associated miRNAs, miR-15a-5ß, miR-17-5p, miR-21-5p and miR-451a, represent themselves as stable miRNAs and were chosen for further research (**a**). Comparison of 5 patients from the Propofol group and 5 patients from Sevoflurane group at time points of awake patients, anesthesia induction and 30 min of surgery (**b**). Numbers in parentheses indicate the number of miRNAs expressed per time point; percentages refer to baseline (awake).

First, the total expression of miRNAs was measured from all 10 patients, independent of randomization (Figure 4a). Here, a total of 192 miRNAs were amplified, with a 12% increase after anesthetic induction and a 5% increase up to 30 min of surgery time relative to baseline. Figure 4b shows the miRNA expressions at different time points for the Propofol and Sevoflurane groups separately.

In the Propofol group, there was a 30% increase in miRNA expression after induction of anesthesia, whereas a slight decrease could be detected in the Sevoflurane group (−6%). Even after 30 min of surgery, there was a 9% increase in miRNAs in the Propofol group compared with baseline. In contrast, miRNAs dropped slightly further to −9% in the Sevoflurane group. A total number of 176 miRNAs in the Propofol group and 159 miRNAs in the Sevoflurane group could have been detected within baseline, after induction of anesthesia and 30 min after the beginning of surgery. As Venn diagrams represent partial quantities, some miRNAs expressed in both groups can overlap. In conclusion, the Propofol group showed an increased particle concentration and expression of EV miRNAs during surgery, whereas the Sevoflurane group showed a nonsignificant decrease in particle concentration and slightly fewer miRNAs.

**Figure 5:**
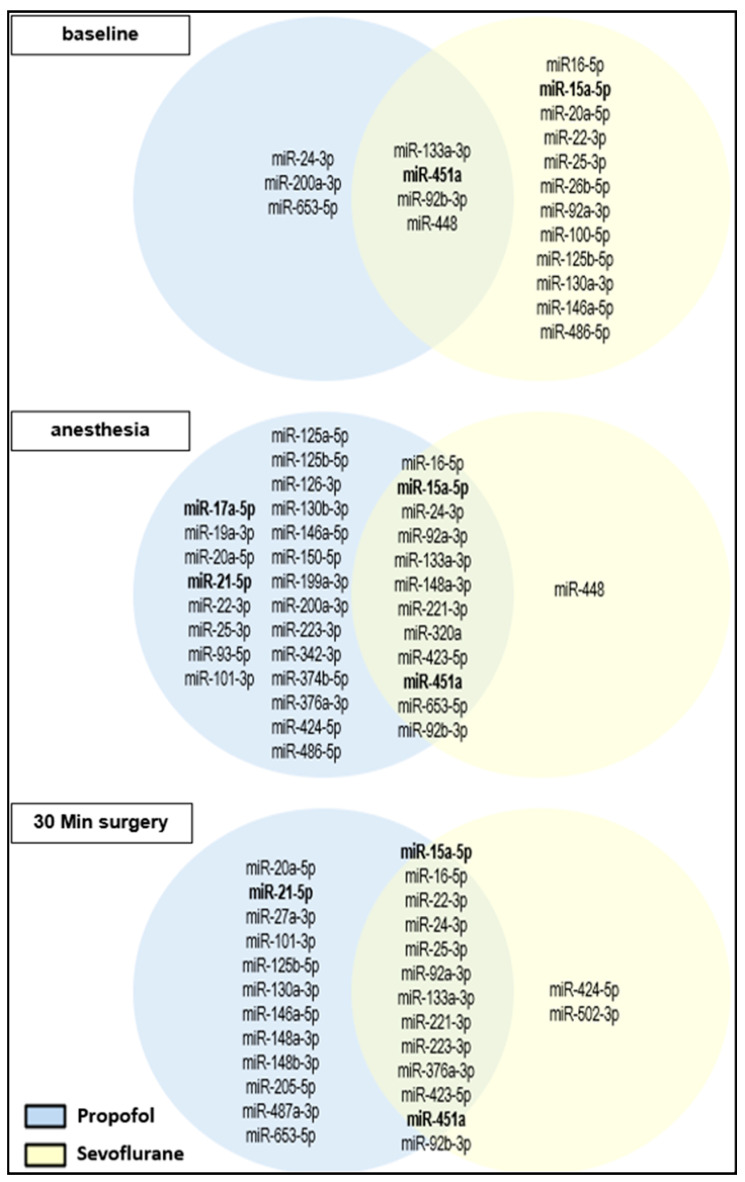
miRNA expression patterns of array studies in Venn diagrams for Sevoflurane and Propofol. Shown miRNAs were expressed within one time point by all patients of one group (Propofol or Sevoflurane). Tumor-associated miRNAs are shown in bold.

Individual miRNAs that were amplified from all patients of each group are presented in Figure 5 with some tumor-associated miRNAs shown in bold.

### 3.5. Detection of Individual miRNAs from EVs with Single Assay Analysis

**Figure 6:**
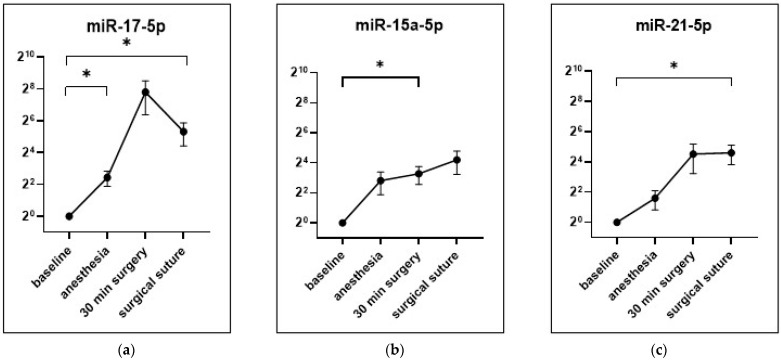
Expression of selected miRNAs. Represented as fold change, relative to basal time point, for the measurement time points of anesthesia induction, 30 min of surgery and suture. Shown are miR-17-5p (**a**), miR-21-5p (**b**), miR-15a-5p (**c**) and mean ± SEM; * *p* < 0.05.

Four tumor-associated miRNAs of the array results, which were expressed in all individuals at all time points, were selected for further investigation. Figure 6 shows the expression fold changes in miR-17-5p, miR-15a-5p and miR-21-5p in all patients after induction of anesthesia and 30 min of surgery and at suture. For miR-17-5p, a significant increase was observed both after induction of anesthesia (fc: 5.39 ± 1.72, *p* = 0.014) and at suture (fc: 39.8 ± 18.3, *p* = 0.042; Figure 6a). MiR-15a-5p showed a significant increase after 30 min of surgery (fc: 9.7 ± 3.82, *p* = 0.027; Figure 6b), whereas miR-21-5p presented an increased expression at suture (24.38 ± 10.24, *p* = 0.029; Figure 6c). Stratification with the anesthetic technique showed no significant difference in expression levels (not shown).

**Figure 7:**
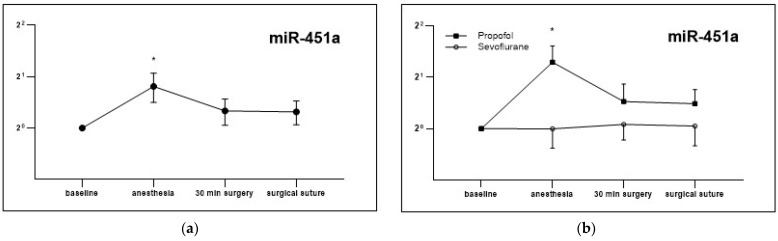
miRNA expression of miR-451a. Represented as fold change, relative to basal time point, for the measurement time points of anesthesia induction, 30 min of surgery and at suture. Shown as the expression for the entire patient population (**a**), as well as differentiated after randomization (**b**), mean ± SEM; * *p* < 0.05.

For miR-451a, an increase in expression was observed during surgery, which was significant after induction of anesthesia (fc: 1.75 ± 0.342, *p* = 0.03; Figure 7a). As miR-451a has antiproliferative effects and inhibits cancer growth in bladder cancer cells in vitro [30], the expression enhancing effect of Propofol on miR-451a should be considered with special attention. When differentiating Propofol and Sevoflurane groups, this increase was due to an expression increase in the Propofol group only (Prop fc: 2.5 ± 0.6, *p* = 0.022; Figure 7b), whereas expression in the Sevoflurane group remained almost identical. Comparison of expression levels of both groups revealed significant differences after induction of anesthesia (*p* = 0.032; Figure 7b). An increased expression of tumor suppressor miR-451a due to Propofol may be a reason for reduced tumor cell viability, which could possibly affect outcome.

## 4. Discussion

EVs are signal mediators involved in numerous processes of carcinogenesis and appear to be differentially affected by anesthetics. For bladder carcinoma patients, anesthetic effects on EVs have not yet been investigated in depth. This work investigated the influence of Propofol and Sevoflurane on EVs and associated miRNA signatures of bladder carcinoma patients undergoing radical cystectomy.

We were able to demonstrate an increase in particle size and concentration during surgery with Propofol anesthesia, whereas in the Sevoflurane group, there was only an initial increase and a significant decrease in particle concentration at suture. In agreement with our findings, Buschmann et al. showed a decrease in particle concentration postoperatively in colon cancer patients, although this had only been significant in the Sevoflurane anesthesia patients [31]. For hepatocellular carcinoma, Propofol has also been shown to increase secretion-of-growth-inhibitory miRNA-bearing EVs from tumor-associated macrophages in in vivo mouse models [32], being consistent with our results. However, other studies demonstrated decreased EV secretion from microglial and endothelial cells as well as a decreased total EV amount following Propofol administration [33,34].

It remains to be discussed what influence the increased EV concentrations in the Propofol group of the present study exert on tumor tissue and the development of micrometastases. It has already been shown that incubation of colon carcinoma cells with sera from colon carcinoma patients who received Propofol anesthesia increased the apoptosis rate compared to Sevoflurane anesthesia [25], which indicates that serum factors are regulated using anesthetics and these might influence tumor growth, with a particular focus on EVs and associated miRNAs [35]. Propofol was shown to exhibit antitumor effects by regulating different miRNAs and lncRNAs and thus influencing important signaling pathways of carcinogenesis [36]. Moreover, Propofol inhibits proliferation, migration and invasion of bladder carcinoma cells through increased micro-RNA expression in vitro, being in line with our results [24]. In addition, Propofol can interfere with carcinogenic signaling pathways such as the hedgehog signaling pathway of bladder carcinoma, thereby exerting anti-oncogenic effects [37,38]. Whether EVs are involved in mediating tumor-inhibitory effects of Propofol has been poorly investigated so far. For hepatocellular carcinoma, EV-mediated tumor growth inhibition induced with Propofol could be demonstrated in an in vivo mouse model. Propofol promoted the release of EV subtypes from tumor-associated macrophages, which could inhibit the invasion and growth of hepatocellular carcinoma cells via the transport of a tumor-suppressive miRNA, miR-142-3p [32]. Meanwhile, interactions between EVs and tumor-associated protective as well as oncogenic macrophages have been more extensively studied and are already being discussed and explored as therapeutic targets [39].

Since surgical stress promotes inflammatory and proangiogenic signaling pathways that favor growth and settlement as metastases of residual tumor cells, and EVs are involved in this process [40], anesthetics could show an impact on EVs that could be involved in the progression of residual tumor cells and thus patient outcome. Although all samples were treated the same way, other drugs or treatments besides the use of Propofol and Sevoflurane could have an influence on extracellular vesicles and associated miRNAs. As miRNAs themselves can be influenced by different medications used during anesthesia [41], other drugs used may influence expression of extracellular vesicles and their cargo. Statistical analyses of other intraoperative-used drugs did not show any significant differences in use of anesthetics. Only the use of noradrenaline was significantly increased in the Sevoflurane group, which may be due to vasodilatation using Sevoflurane. While EVs may regulate blood pressure in animal models [42], noradrenaline administration might be a confounder. Sevoflurane led to a reduced concentration of EVs during surgery; therefore, one may speculate that an increased EV concentration may lead to less suppressed vasodilatation and favor hypotension, which leads to increased noradrenaline use, but this is rather speculative. In statistical analyses, other drugs did not show any other significant differences between the Propofol and Sevoflurane groups, indicating that there are no significant influences of other drugs on EVs. Due to the relatively small case number of this pilot study, it was not possible to build absolutely homogeneous groups regarding patient and intraoperative characteristics, but our results do not show any hints on specific interaction between EVs and other drugs.

To further analyze the potential role of EVs on tumor progression, EV-dependent miRNAs were subsequently investigated. Buschmann et al. already found in a non-randomized pilot study of colorectal cancer patients that EV-associated miRNAs are differentially regulated depending on the anesthetic regime [31]. For bladder cancer patients, we could show an increased expression of EV-dependent miRNAs in the Propofol group, whereas the expression patterns slightly decreased in the Sevoflurane group (Figure 4B).

Array analyses showed expression of different miRNAs expressed by bladder cancer patients during surgery (Figure 5). Remarkably, many detected miRNAs are already known to be associated with bladder cancer such as miR-223 or miR-221, which are upregulated in urothelial carcinoma tissue compared to normal mucosa [43]. Other detected miRNAs such as miR-93 or miR-200 have been investigated as biomarkers to detect bladder cancers by using miRNAs obtained in the urine [44]. miRNAs appearing as possible biomarkers in the tissue and urine of bladder cancer patients could also be found in plasma. This indicates an interaction between possible regulators of bladder cancer, carcinogenesis and anesthetics. As array results showed differences in miRNA expression, further analyses of some miRNAs were carried out afterwards.

To test whether EV-miRNA expression changes during surgery, four specific tumor-associated miRNAs [43,45] were selected and measured as individual assays from all patients (Figure 6). For miR-17-5p, miR-21-5p and miR-15a-5p, a significant increase in expression was detected. However, this was independent of the anesthetic used, indicating that surgery itself promotes expression changes of specific tumor-associated EV-miRNAs.

For miR-451a, Propofol resulted in expression changes after induction of anesthesia while Sevoflurane anesthesia had no effect, although Propofol was used for anesthesia induction in both groups (Figure 7). While Propofol-mediated effects on EV release seem to be dose-dependent [35], higher doses of Propofol in the Propofol group or a potentially inhibitory effect of Sevoflurane on miR-451a may explain the results.

Overexpression of miR-451a in urinary bladder carcinoma cells showed antiproliferative and invasion inhibitory effects in vitro, suggesting that miR-451a acts as a tumor suppressor for urothelial cells and reduces their cell viability [30]. For gastric cancer cells, anti-oncogenic effects were measured after Propofol administration via increased miR-451a expression. In this publication, a direct regulation of miR-451 with Propofol was shown. MiR-451 has commonly low expressions in gastric cancer cells but increased miR-451a is associated with reduced tumor cell growth by regulating matrix metalloproteinases. Propofol enhances miR-451 expression and thereby reduces MMP expression, which explains the tumor-suppressive effects of Propofol on a biomolecular level [46]. In addition, in colorectal, non-small-cell lung and renal cell carcinomas; glioblastoma; and other carcinomas, miR-451 functions as a tumor suppressor and may be used as a diagnostic biomarker in the future [47].

Additional research of the effects of Propofol on miR-451a in bladder carcinoma cell lines and cell viability will be needed to further elucidate the results. In the future, knowledge of mechanisms of action of anesthetics on tumor-associated miRNAs may provide mechanistic insights into potential anesthetic-dependent outcome differences.

If the choice of anesthesia affects tumor-inhibitory signaling pathways in patients that could prevent the development of metastases and recurrences via EVs and associated miRNAs, this possible therapeutic potential should be exploited and explored further. EVs themselves are already being explored as potential drug delivery vehicles for cancer therapy [48,49] while Propofol is under investigation as a chemosensitizer [36]. If Propofol was demonstrated to result in EV-modulated tumor inhibition in the surgical setting, this would be a simple and desirable way to improve patient outcome.

Our results suggest that an increased concentration of EVs as well as elevated levels of tumor-suppressive miR-451a may be an explanation for reduced tumor cell viability after the use of Propofol. As this study was an observational study, we cannot surely distinguish whether an increased count of extracellular vesicles or the altered miRNA expression itself is responsible for tumor progression. As altered miRNA expression also depends on the amount of circulating EVs, and EVs have been shown to play a crucial role in tumor progression [40], we suggest that EVs alter tumor progression rather than one single miRNA. miRNAs are important modulators of gene expression and can regulate cell growth, but one should keep in mind that EVs not only transport one single miRNA but rather many miRNAs next to mRNA, DNA and proteins. These all could possibly influence cell growth.

To elucidate our results further, in vitro and in vivo studies are needed to validate the effect of EVs after Propofol on bladder cancer cells. Additionally, pathway analyses of found miRNAs may give a better explanation of the influence on tumor cell growth.

Various interactions between anesthetics and cancer progress through regulation of associated miRNAs and their controlling signaling pathways are already known. While Propofol seems to have antitumor effects and Sevoflurane enhances tumor progress, it is important to mention that effects of anesthetics on cancer progression through miRNA regulation are dependent on cancer cell type. The idea of analyzing miRNA profiles of special miRNA clusters with a bioinformatic pathway analysis may enhance the knowledge of different anesthetics that have been used during surgery [41].

This was a monocenter study in which, despite of randomization, more women were included in the Sevoflurane group than in the Propofol group. Both groups received Propofol for induction of anesthesia, which could possibly influence the effects of Sevoflurane. Therefore, the use of Propofol for induction of anesthesia in the Sevoflurane group may have potentially influenced particle size and concentration at this time point. Since Propofol contains lipids, it could be considered a confounder for particle size and concentration measurements. However, particle sizes of Propofol were shown to be clearly above the size range of exosomes [50,51]. Therefore, an influence of Propofol on particle size measurements seems to be unlikely due to the much smaller average particle size in our study compared to the expected range of Propofol. Regarding particle concentrations, studies in mice have shown a Propofol plasma concentration of 25% after 30 min of the original concentration [52], and concentration measurements in humans after a single administration of Propofol revealed a half-life in blood plasma of 2.3 ± 0.7 min [53]. Therefore, no influence of Propofol on particle sizes and concentrations 30 min after incision is to be expected in the Sevoflurane group. Furthermore, comparison of particle sizes after induction of anesthesia of both randomized groups showed a significant difference with mean particle sizes of 132 ± 11.5 nm (Prop) versus 120 ± 8.87 nm (Sevo; *p* = 0.0002). If Propofol actually had an impact on the results of the Sevoflurane group, comparable particle sizes would have been expected. Accordingly, the influence of the single Propofol administration in the Sevoflurane group seems to be negligible and the measurement results can indeed be interpreted as a function of the different anesthetics. As an alternative, another intravenous drug, such as ketamin, ethomidat or thiopenthal, could have been used for anesthesia induction in the Sevoflurane group. These drugs could also have influenced EV expression and possibly may have influenced the effect of Sevoflurane on EVs, so our study protocol enables ruling out that other drugs influenced EV expression. However, using a polymer-based method, other particles and non-EVs could potentially have been co-measured, therefore indicating a methodological issue. Regarding generalizability of these results, it is to be considered that both EVs and miRNAs can vary substantially within different persons and different carcinomas. For further studies, it is recommended to test a larger patient collective. As we already discussed, the results should be investigated further in vitro and in vivo. In order to gain insights into overall survival and cancer-specific survival between patients who received Sevoflurane or Propofol, a long-term follow up may improve an understanding on the clinical impact EVs can have.

## 5. Conclusions

Here, we could demonstrate that EV miRNA signatures in urinary bladder carcinoma patients change depending on the anesthetic procedure during radical cystectomy. Propofol anesthesia leads to increased EV size and concentration and EV-associated miRNA expression, whereas Sevoflurane only slightly alters or decreases EV concentration and miRNA expression. Moreover, tumor-suppressive miR-451a increased intraoperatively in the Propofol group only. This may provide an explanatory approach to the decreased tumor cell viability under Propofol anesthesia. As a prospective randomized controlled trial, the results of this work could serve as the basis for further studies exploring the effect of anesthetics on EVs and tumor growth. 

## Figures and Tables

**Table 1:** Demographics and intra- and postoperative characteristics. Demographic data from all of the patients of the study. Postoperative data refer to the next morning after surgery. M ± SD, mean and standard deviation; Md (IQR), median with interquartile range; MAC, minimal alveolar concentration; BMI, body mass index; ASA, American society of anesthesiology; UICC, Union for international cancer control; TURBT, transurethral resection of bladder tumor; T, local tumor stage, N; N, nodal tumor stage; M, presence of metastatic disease; *p*, histopathologically confirmed cancer; y, neoadjuvant chemotherapy.

	Total	Sevoflurane	Propofol	*p* Value
Number	51	25	26	
Age (years), M ± SD	67.5 ± 9.19	66.5 ± 10.1	68.5 ± 8.25	0.451
Sex (male/female), n (%)	41(80)/10(20)	17(68)/8(32)	24(92)/2(8)	0.038
BMI (kg/m^2^), Md (IQR)	28.1 (5.51)	28 (5.93)	28.3 (6.49)	0.445
ASA-Score, n (%)				
I	1(2)	1(4)	0	
II	28(55)	16(64)	12(46)	
III	22(43)	8(32)	14(54)	
UICC BC, n (%)				
0is	9(17.5)	2(8)	7(27)	
I	7(14)	4(16)	3(11)	
II	9(17.5)	5(20)	4(15)	
IIIa	13(25)	7(28)	6(23)	
IIIb	2(4)	1(4)	1(4)	
IVa	1(2)	1(4)	0	
pT0N0M0 after TURBT, n (%)	3 (6)	1 (4)	2 (8)	1000
ypT0N0M0, n (%)	7 (14)	4 (16)	3 (12)	1000
Neoadjuvant therapy, n (%)	19 (37)	9 (36)	10 (38)	0.962
Packyears, M ± SD	32 ± 22.6	35.4 ± 26.3	29 ± 19.2	0.466
Creatinine (mg/dL), Md (IQR)	1.0 (0.2)	1.0 (0.3)	1.0 (0.2)	0.985
Hemoglobin (mg/dL), Md (IQR)	13.3 (2.95)	13.5 (3.5)	13.0 (3.2)	0.479
Atrial fibrillation, n (%)	6 (12)	1 (4)	5 (19)	0.191
Arterial hypertension, n (%)	37 (73)	18 (72)	19 (73)	0.931
Diabetes, n (%)	6 (12)	3 (12)	3 (11.5)	1.000
Intraoperative data				
Open/robotic surgery, n (%)	33(65)/18(35)	16(64)/9(36)	17(65)/9(35)	0.918
Duration of surgery (Min), M ± SD	343 ± 79.2	343 ± 92.5	342 ± 65.8	0.971
Propofol (mg), Md (IQR)	1595 (2956)	180 (40)	3075 (1819)	<0.001
MAC, M ± SD		0.923 ± 0.164		
Fentanyl (mg), Md (IQR)	250 (50)	250 (100)	250 (100)	0.454
Noradrenaline (mg), Md (IQR)	2.3 (3.47)	3.2 (2.85)	1.83 (3.18)	0.030
Blood loss (mL), Md (IQR)	600 (613)	500 (325)	800 (750)	0.235
Postoperative data				
Creatinine (mg/dL), Md (IQR)	1 (0.3)	1 (0.3)	1 (0.4)	0.732
Hemoglobin (g/dL), Md (IQR)	9.4 (2.5)	9.1 (2.8)	9.55 (1.7)	0.712
Rest pain, Md (IQR)	1 (2)	0 (3)	1 (2)	0.992
Length of stay (days), Md (IQR)	13 (2)	12 (3)	13 (2)	0.425

## Data Availability

The data presented in this study are available on request from the corresponding author. The data are not publicly available due to privacy.

## Supplementary Materials

The following supporting information can be downloaded at: https://www.mdpi.com/article/10.3390/cells12202503/s1, Supplemental digital content S1: Extraction of extracellular vesicles. Experiment protocol using ExoQuick-TCTM Exosome Precipitation Solution and PD SpinTrapTM G-25 (Cytiva, Marlborough, USA) to extract extracellular vesicles from plasma samples. Supplemental digital content S2: Demographics for Array Analysis. Data from the ten patients for miRNA array analysis. M ± SD, mean and standard deviation; Md (IQR), median with interquartile range; MAC, minimal alveolar concentration; BMI, body mass index; ASA, American society of anesthesiology.
