# Supplementary material for: Influence of the Anesthetic Technique on Circulating Extracellular Vesicles in Bladder Cancer Patients Undergoing Radical Cystectomy: A Prospective, Randomized Trial
Source: Cells. 2023 Oct 23;12(20):2503. doi: 10.3390/cells12202503 (PMC10605791; doi:10.3390/cells12202503)
Supplement: Supplementary file 1 [file cells-12-02503-s001.zip › ExoRC_cells_suppdigcont1_final.pdf]

**Supplemental digital content S1: Extraction of extracellular vesicles.** Experiment protocol using ExoQuick-TC$^{TM}$ Exosome Precipitation Solution and PD SpinTrapTM G-25 (Cytiva, Marlborough, USA) to extract extracellular vesicles from plasma samples.

- Thaw ExoQuick-TC$^{TM}$ Exosome Precipitation Solution (System Biosciences, Palo Alto, USA), which must be at room temperature for processing
- Thaw EDTA plasma sample on ice and vortex well after defrosting
- Centrifuge EDTA plasma sample for 3 Min, 4°C, 3000 G
- Take 450 µl from the supernatant and put it into a 1,5 ml LoBind Eppendorf tube (Eppendorf AG, Hamburg, Germany) and label it, discard the pellet
- Centrifuge supernatant from EDTA plasma sample again for 15 Min, 4°C, 10.000 G
- While centrifuge is running: prepare ExoQuick-TC$^{TM}$ Exosome Precipitation Solution
    - Add 200µl of ExoQuick-TC$^{TM}$ Exosome Precipitation Solution slowly to a 2 ml LoBind tube (be careful, exosome precipitation solution is very viscous, pipette slowly)
    - Add 400 µl of 1:100 diluted 2-(4-(2-hydroxyethyl)-1-piperazinyl)-ethanesulfonic acid (HEPES)/ sodium chloride mixture to the exosome precipitation solution and vortex shortly
- Add 400µl oft the supernatant from EDTA plasma sample to the 2 ml LoBind with exosome precipitation solution and vortex shortly
- Place in the refrigator overnight (18 hours)
- Next morning: centrifuge tube for 30 Min, 4°C, 1500 G
- Remove 1ml supernatant and put it in 1.5 LoBind tube for other analyses
- Discard 170 µl of the supernatant, don't touch the pellet
- Centrifuge 5 Min, 4°C, 1500 G
- Remove supernatant from pellet
- Add 170µl of HEPES/ sodium chloride mixture to the pellet
- Vortex for 10 sec, let stand for 5 Min
- Repeat until pellet is dissolved
- Prepare PD SpinTrapTM G-25 (Cytiva, Marlborough, USA) for filtering pellet and removing polymers
    - Vortex filter (lid and bottom)
    - Open lid slightly, snap off bottom cap
    - Insert filter into collection tube
    - Centrifuge empty filter 1 Min, 20°C, 800G and remove storage solution
    - Equilibrate the filters:

- add 400 µl HEPES/ sodium chloride mixture on the filter and centrifuge 1 Min, 20°C, 800 G
- discard flow-through, replace filter and repeat procedure four times
- after rinsing five times in total, discard collection tube and set filter to 1.5 LoBind tube

- sample application and elution: put 200µl of dissolved exosome suspension in the center of the filter
- centrifuge 2 Min, 20°C, 800G, discard the filter
- the tube now contains the filtered exosome suspension
- put 170 µl of the exosome solution into 2ml LoBind tube for RNA isolation

A. B. C.

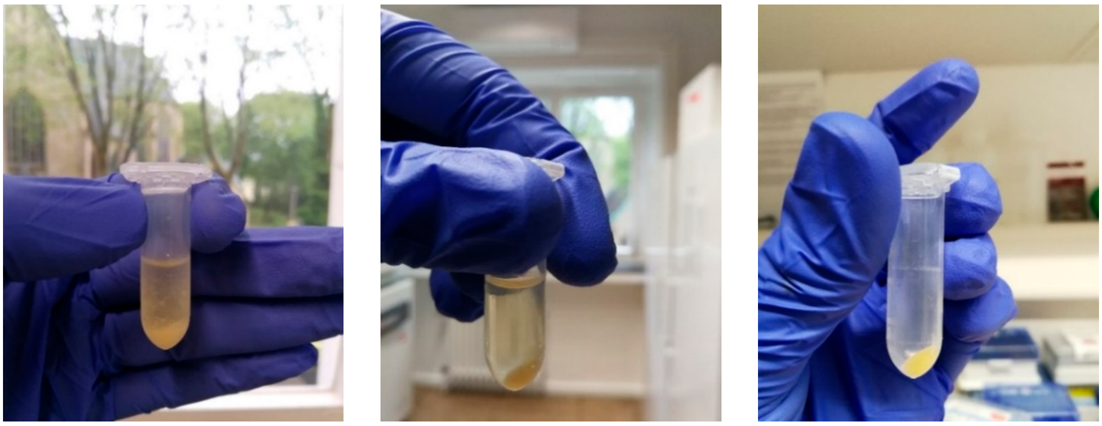

Extraction of extracellular vesicles using ExoQuick-TCTM Exosome Precipitation Solution. Precipitated EVs after 18 h incubation at 4°C are shown (A), as well as the EV pellet after centrifugation with EV-free EDTA plasma supernatant (B) and the EV pellet for further processing (C).
