# Supplementary material for: Influence of the Anesthetic Technique on Circulating Extracellular Vesicles in Bladder Cancer Patients Undergoing Radical Cystectomy: A Prospective, Randomized Trial
Source: Cells. 2023 Oct 23;12(20):2503. doi: 10.3390/cells12202503 (PMC10605791; doi:10.3390/cells12202503)
Supplement: Supplementary file 1 [file cells-12-02503-s001.zip › ExoRC_cells_suppdigcont2_final.pdf]

Supplemental digital content S2: Demographics for Array-Analysis.

Data from the ten patients for miRNA array analysis. M ± SD, mean and standard deviation; Md (IQR), median with interquartile range; MAC, minimal alveolar concentration: BMI, Body mass index; ASA, American society of anesthesiology

	Total	Sevoflurane	Propofol	P Value
Patient characteristics				
number	10	5	5	
age (years), M ± SD	64.3 ± 8.81	64.8 ± 11.2	63.8 ± 7	0.880
sex (male/female), n (%)	8(80) / 2(20)	3(60) / 2(40)	5(100) / 0	0.444
BMI (kg/m^2), M ± SD	29 ± 4.57	27.5 ± 2.98	30.6 ± 5.65	0.307
ASA-Score (I/II/III), n (%)	4(40) / 6(60)	3(60) / 2 (40)	1(20) / 4(80)	0.524
Neoadjuvant therapy, n (%)	7(70)	3 (60)	4(80)	1.000
Packyears, M ± SD	27.1 ± 23.7	14 ± 15.2	40.2 ± 24.6	0.077
Creatinine (mg/dl), M ± SD	0.96 ± 0.14	1.02 ± 0.11	0.9 ± 0.16	0.201
Hemoglobin (mg/dl), M ± SD	12.2 ± 1.14	11.8 ± 1.31	12.7 ± 0.83	0.221
Blood sugar (mg/dl), M ± SD	133 ± 31.6	127 ± 34.1	139 ± 31.6	0.586

	total	Sevoflurane	Propofol	P Value
Intraoperative data				
Open/robotic surgery, n (%)	6(60) / 4(40)	2(40) / 3(60)	4(80) / 1(20)	0.524
Duration of surgery (Min), M ± SD	358 ± 67.3	356 ± 83.9	361 ± 55.9	0.908
Fentanyl (µg), Md (IQR)	300 (63)	300 (75)	300 (125)	0.841
Propofol induction (mg), M±SD	280 ± 58.7	194 ± 34.4	222 ± 73.6	0.463
Propofol total (mg), Md (IQR)	1454 (2915)	180 (55)	2775 (1649)	0.008
MAC, M ± SD		0.82 ± 0,06		
Ropivacaine (mg), Md (IQR)	40 (11)	40 (38)	40 (13)	0.421
Jonosteril (ml), M±SD	3805 ± 1230	3200 ± 1303	4410 ± 889	0.125
Noradrenaline (mg), Md (IQR)	4.54 (2.93)	2.16 (2.87)	4.47 (2.84)	0.347
Blood loss (ml), M±SD	635 ± 411	490 ± 283	780 ± 497	0.290
Postoperative data				
Creatinine (mg/dl), M±SD	0.97 ± 0.14	1.02 ± 0.13	0.92 ± 0.15	0.290
Hemoglobin (g/dl), M±SD	9.13 ± 0.77	8.76 ± 0.86	9.5 ± 0.49	0.135
Hospital days, Md (IQR)	12.5 (3)	12 (3)	13 (4)	0.130
